# In response to Taekman: “On the Prior Use of High-Fidelity Simulation to Improve Clinical Trial Development and Implementation”

**DOI:** 10.1017/cts.2025.10216

**Published:** 2026-01-22

**Authors:** Ronnie Guillet, Rita Dadiz

**Affiliations:** Division of Neonatology, Department of Pediatrics, Golisano Children’s Hospital, University of Rochester Medical Center, Rochester, NY, USA

We greatly appreciate Dr Taekman’s contacting us to describe the work he and his team published 15-20 years ago. Although we had independently searched the literature, neither Dr Dadiz nor I became aware of his manuscripts while working on ours. As Dr Taekman noted, our findings of the potential benefits of utilizing simulation to prepare for clinical trial initiation closely mirror those revealed by his team’s research, although we were unable to test our hypotheses to verify them in the context of an ongoing clinical trial.

Even though the use of simulation has grown tremendously in the past 20 years, as evidenced by the thousands of publications describing the many contexts in which it may accelerate progress in a variety of fields, it still is not routinely employed to facilitate the initiation of complex clinical trials. While manuals of procedures are reviewed with clinical research teams and limited simulation exercises may be conducted to standardize procedures, simulation has not been embraced as described in Dr Taekman’s and our papers. We suggest that although the evidence presented by Dr Taekman is convincing, a true test of the usefulness of simulation would be a randomized trial of this technique when a multicenter clinical trial is initiated, with half of the participating centers using simulation and the other half using more “traditional” onboarding practices.

We share Dr Taekman’s sentiment and hope researchers find the use of simulation in these collective articles instructive as they develop and initiate their own clinical trials.

